# Epigenome-wide DNA methylation analysis of whole blood cells derived from patients with GAD and OCD in the Chinese Han population

**DOI:** 10.1038/s41398-022-02236-x

**Published:** 2022-11-07

**Authors:** Liangkun Guo, Zhaojun Ni, Guiming Wei, Weiqiu Cheng, Xuebing Huang, Weihua Yue

**Affiliations:** 1 Institute of Mental Health, Peking University Sixth Hospital, Beijing, 100191 China; 2 National Clinical Research Center for Mental Disorders (Peking University Sixth Hospital), Beijing, 100191 China; 3 NHC Key Laboratory of Mental Health, & Research Unit of Diagnosis and Treatment of Mood Cognitive Disorder, Chinese Academy of Medical Sciences, Beijing, 100191 China; 4 Department of Neurology, Shandong Daizhuang Hospital, 272051 Jining, Shandong China; 5 NORMENT, Division of Mental Health and Addiction, Oslo University Hospital & Institute of Clinical Medicine, University of Oslo, Oslo, Norway; 6 PKU-IDG/McGovern Institute for Brain Research, Peking University, Beijing, 100871 China; 7 Chinese Institute for Brain Research, Beijing, 102206 China

**Keywords:** Psychiatric disorders, Epigenetics in the nervous system

## Abstract

Generalized anxiety disorder (GAD) and obsessive-compulsive disorder (OCD) had high comorbidity and affected more than 44 million people around the world leading to a huge burden on health and economy. Here, we conducted an epigenome-wide DNA methylation study employing 93 patients with GAD, 65 patients with OCD, and 302 health controls, to explore epigenetic alterations associated with the onset and differences of GAD and OCD. We identified multiple differentially methylated positions (DMPs) and regions (DMRs): three DMP genes included *RIOK3* (cg21515243, *p* = 8.00 × 10^−10^), *DNASE2* (cg09379601, *p* = 1.10 × 10^−9^), and *PSMB4* (cg01334186, *p* = 3.70 × 10^−7^) and two DMR genes *USP6NL* (*p* = 4.50 × 10^−4^) and *CPLX1* (*p* = 6.95 × 10^−4^) were associated with the onset of GAD and OCD; three DMPs genes included *LDLRAP1* (cg21400344, *p* = 4.40 × 10^−12^), *ACIN1* (cg23712970, *p* = 2.98×10^−11^), and *SCRT1* (cg25472897, *p* = 5.60 × 10^−11^) and three DMR genes *WDR19* (*p* = 3.39 × 10^−3^), *SYCP1* (*p* = 6.41 × 10^−3^), and *FAM172A* (*p* = 5.74 × 10^−3^) were associated with the differences between GAD and OCD. Investigation of epigenetic age and chronological age revealed a different epigenetic development trajectory of GAD and OCD. Conclusively, our findings which yielded robust models may aid in distinguishing patients from healthy controls (AUC = 0.90–0.99) or classifying patients with GAD and OCD (AUC = 0.89–0.99), and may power the precision medicine for them.

## Introduction

Generalized anxiety disorder (GAD) and obsessive-compulsive disorder (OCD), affected more than 44 million people around the world [1]. Both GAD and OCD can cause marked anxiety or distress on patients: GAD makes patients experience worry and fear about everyday situations frequently, intensively, excessively, and persistently, that interfere the daily life and are hard to control; OCD makes patients experience recurrent and persistent thoughts, urges or images which were intrusive and unwanted [2]. Meta-analysis indicated the direct cost and indirect cost of GAD and OCD achieved the 2.08 and 0.22% of gross domestic product [3], respectively. GAD and OCD had been proved to be associated with environmental factors like prenatal events [4], urban environment [5], and drug abuse [6]. DNA methylation (DNAm), a major epigenetic modification in humans, regulates the expression of genes and alters the trait without alteration in DNA sequence. DNAm changes [7–9] were observed in patients with GAD or OCD, which indicated their pathological role in them. There is a challenge in the diagnoses because of the high risk of co-morbidity and common symptoms among GAD and OCD [10, 11]. To date, studies only focused on one disease, and the similarities and differences of epigenetic structures among them hadn’t been elucidated yet.

In this study, we employed 93 patients with GAD, 65 patients with OCD, and 302 health controls (HC) to investigate the DNAm alteration associated with the onset of GAD and OCD and the differences and similarities in DNAm structure between them. In addition, we will testify if the observations can be a diagnosis tool aiming in aiding the precision medicine of GAD and OCD.

## Materials and methods

### Participants

In this study, a total of 158 drug-naïve and first-episode patients (93 GADs and 65 OCDs) were recruited in Peking University Sixth Hospital from 2013 to 2021. The age (mean ± S.D) of GAD and OCD was 36.94 ± 12.07 and 29.31 ± 10.06, respectively. The ratio of sex (male:female) of GAD and OCD was 39:54 and 43:22, respectively. Diagnosis and collection of blood samples were conducted by the clinical research physicians from Peking University Sixth Hospital. All patients were diagnosed with GAD or OCD according to the criteria of the Diagnostic and Statistical Manual of Mental Disorders, fourth edition (DSM-IV).

We recruited a total of 302 healthy participants in Beijing. The age (mean ± S.D) of healthy control was 24.47 ± 3.29. The ratio of sex (male:female) of healthy control was 149:153. We included participants if they were (1) Chinese of Han ancestry (including the participants and their parents); (2) age ranging from 18 to 45; (3) no current or history of neurological or psychiatric disease met the criteria of Diagnostic and Statistical Manual of Mental Disorders, 4th Edition, Text Revision (DSM-IV-TR), assessed by psychiatrists using the Structured Clinical Interview for DSM-IV-TR Axis I Disorders, Non-patient Edition (SCID-I/NP). We excluded participants if they had (1) a history of psychiatric or neurological diseases and substance abuse or dependence (including the participants and their parents); had a history of loss of consciousness for more than 5 min; (3) are pregnant or lactating women or women planning to become pregnant.

The demography of participants and statistics of clinical information can be found in Table 1. This study was approved by the Institutional Review Boards of Peking University Sixth Hospital and the written informed consent was obtained from each participant.

**Table 1:** Demography.

	Cases (*n* = 158)		HC (*n* = 302)
	GAD (*n* = 93)	OCD (*n* = 65)	
Age (mean±S.D)	33.80 ± 11.87^a^		24.47 ± 3.29^a^
	36.94 ± 12.07^b^	29.31 ± 10.06^b^	
Sex (female, %)	76 (48.10%)		153 (50.66%)
	54 (58.06%)	22 (33.84%)	

^a^Wilcoxon test, age_cases_ vs age_HC_, *p* = 1.47 × 10^−16^; ^b^Wilcoxon test, age_GAD_ vs age_OCD_, *p* = 4.13 × 10^−5^.

### Epigenome-wide DNA methylation profiling and quality control

Genomic DNA was extracted with the QIAamp DNA Mini Kit (QIAGEN, Hilden, Germany) from whole blood and treated with sodium bisulfite following standard procedures. Genome-wide DNA methylation was assessed using Infinium HumanMethylation450 BeadChip and EPIC BeadChip. Firstly, we tried to filter samples with at least 5% of the probes that did not pass a 0.05 detection *P* value threshold, and no sample was removed. We then filtered the probes with >0.01 detection P value in more than 5% samples and the probes with <3 bead count in at least 5% samples, and then we filtered probes with annotated SNPs together or with probes located on sex chromosomes or align to multiple locations as identified in Nordlund et al [12]. Beta values (ranging from 0 to 1) were then generated to represent methylation ratios at a given CpG site, and higher beta values indicate higher methylation levels. Technical differences between two different probe types were then normalized by beta-mixture quantile normalization method (BMIQ) [13] as implemented in the “ChAMP” R package [14]. After the processing pipeline, 375,546 probes remained for the following analyses.

### Adjustment of confounders

We used champ.runCombat function to conduct the correction of batch effect. Cell proportion was estimated by DNA Methylation Age Calculator. We included sex, age, blood cell proportion, and principal components that explain 95% of the variance in the linear model by R package limma to reduce the potential confounding.

### Identification of differentially methylated positions

Differentially methylated position (DMP) analysis was performed by “ChAMP.DMP” function from ChAMP package in R. Multiple testing was adjusted using the Benjamini and Hochberg correction, with the significance threshold set at an adjusted *p*-value < 0.05.

### Identification of differentially methylated regions

Differentially methylated region (DMR) analysis was performed to detect differentially methylate with “ChAMP.DMR” function from ChAMP package in R and “Bumphunter” algorithm with parameters as (1) 1000 times of bootstrapping for reducing the bias in sampling; (2) beta value smoothing was performed for each CpG site [14]. *P* values were corrected by the Benjamini–Hochberg method, with the threshold set at an adjusted *p*-value < 0.05. For each DMR, a minimum number of three consecutive CpG sites were required to constitute a DMR.

### Gene ontology analysis

Gene ontology (GO) analysis for DMP-annotated and DMR-annotated genes was conducted by g:Profiler [15]. We used all known genes as reference for the GO enrichment. For each GO term with the *p-*value adjusted by Benjamini and Hochberg that was less than 0.05 was considered significant.

### Epigenetic clock calculation

We used epigenome-wide methylation data to calculate the epigenetic clocks by DNA Methylation Age Calculator (https://dnamage.genetics.ucla.edu). The data for calculation are not normalized by our methylation profiling pipeline and are normalized by the DNA methylation Age Calculator. We use the age acceleration residual, the recommended epigenetic clock measurements [16], to estimate the epigenetic clock profile.

### Evaluation of blood-brain correlation in DNA methylation

We used the online database, Blood-Brain DNA Methylation Comparison Tool (https://epigenetics.essex.ac.uk/bloodbrain/) which included the whole-genome wide methylation data of peripheral blood and brain tissues (prefrontal cortex, entorhinal cortex, superior temporal gyrus, and cerebrum) from the matched individuals, to evaluate the brain-blood correlation. The online database used the Pearson correlation to measure the coefficient of DNA methylation between brain tissues and peripheral blood, *p*-value < 0.05 was considered as the significant correlation.

### Development of diagnostic and distinguish models

The whole model development pipeline was under the machine learning framework provided by R package caret. We used the methylation level of CpG sites which were from the differential methylation analysis (1081 CpG sites for the case-control study and 398 CpG sites for the GAD vs OCD study) and the machine learning algorithm eXtreme Gradient Boosting (xgBoost) to develop the model. We used the following preprocessing procedures for model development: (1) we shuffled all samples and randomly separated them into a training dataset and test dataset with the ratio of 7:3; (2) we scaled, centered, and Box-cox transformed the training dataset, store the data distribution of training dataset, and applied the data distribution into test dataset (to avoid the data leakage); (3) we used 10-fold cross-validation to build the model and to avoid the underfitting or over-fitting; (4) we used the random search to optimize hyper-parameters (e.g., gamma, max depth of the tree, drop rate, etc.), relied on the area under the curve (AUC) to evaluate the model performance, and select the model which was with highest AUC as the ultimate model.

### Statistics

The statistical power of the sample size was estimated by G*power software [17] (version 3.1) under the models of Pearson correlation, ANOVA, and t-test, which can be found in Supplementary figure S1. Statistical analysis was conducted in R 4.1.1. We used Pearson correlation to estimate the correlation coefficient between two variables. A threshold of *p*-value < 0.05 was considered as a significant correlation. We used the Wilcoxon test to compare the difference in mean between two groups and used the Kruskal–Wallis test to compare the difference in mean between three groups, a threshold of *p*-value < 0.05 was considered as the significant difference.

### Data deposition

The DNA methylation data of this study can be accessed on Peking University Open Research Data Platform (10.18170/DVN/ORDT4O).

## Results

### Demography of participants and study design

Table 1 described the demographic information of our participants. We included 93 participants with GAD, 65 participants with OCD, and 302 healthy people (HC). The sample size provided enough statistical power for the following analyses (see Supplementary Fig. S1).

We used the combination of GAD and OCD (cases, all were drug-naive) and the HC to conduct a case-control study investigating the DNA methylation change. In this setting, the age (mean ± S.D) were 33.80 ± 11.87 and 24.47 ± 3.29 in cases and HC, respectively, and there is a significant difference in age between cases and HC (Wilcoxon test, age_cases_ vs age_HC_, *p* = 1.47 × 10^−16^) suggesting a bias of age. Besides, there were 76(48.10%) and 153(50.66%) females in cases and HC, respectively.

Another study was conducted between patients with GAD and with OCD (GAD vs OCD). In this study, the age (mean ± S.D) were 36.94 ± 12.07 and 29.31 ± 13.06 in GAD and OCD, respectively, and there is a significant difference in age between cases and HC (Wilcoxon test, age_GAD_ vs age_OCD_, *p* = 4.13 × 10^−5^) indicating the confounding from age. There were 54(48.10%) and 22(33.84%) females in cases and HC, respectively, which suggested a bias in sex.

Conclusively, the statistical result of demography indicated the potential confounding of age and sex, therefore, as we mentioned in the method section, the adjustment for the confounding factors was applied to the methylation data before the analyses were carried out.

### Differential methylation analyses in the case-control study

**Fig. 1:**
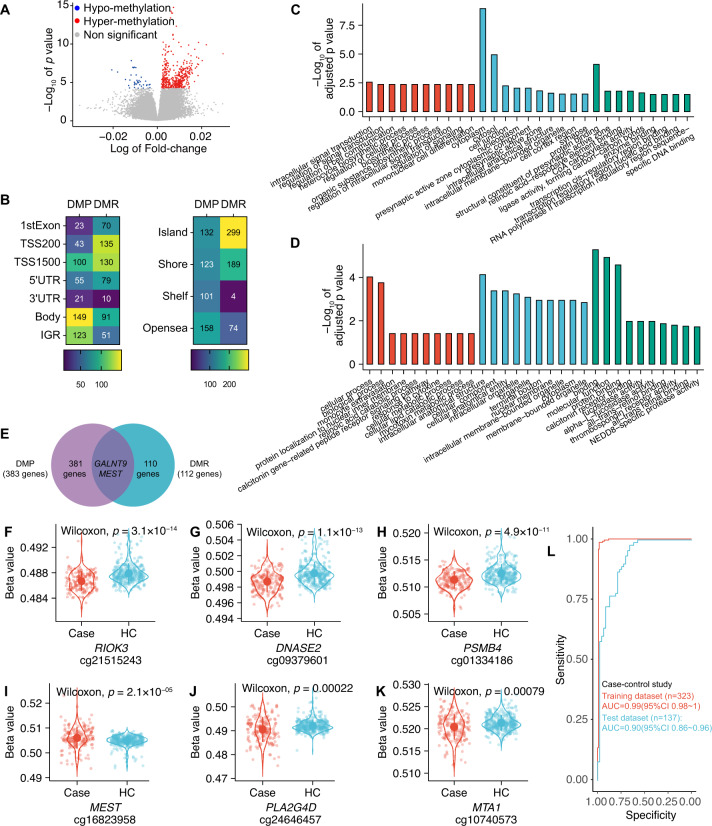
Differential methylation analyses in the case-control study. **A** Volcano plot for differentially methylated positions (DMPs). Gray points represented the nonsignificant DMP; red and blue points represented the significant DMPs with hyper or hypo-methylation (case vs control), respectively. **B** Heatmap plots, the left heatmap described the regions of CpG sites: the detected DMPs were mainly enriched in regions as body (149 CpG sites [28.99%]), IGR (intergenic region, 123 CpG sites [23.93%]), and TSS1500 (100 CpG sites [19.46%]), and were less enriched in TSS200 (43 CpG sites [8.37%]), 5’UTR (55 CpG sites [10.7%]), 3′UTR (21 CpG sites [4.09%]) and 1st exon (23 CpG sites [4.47%]); The CpG sites from DMRs were mainly from the regions as TSS1500 (130 CpG sites [22.97%]), TSS200 (135 CpG sites [23.85%]), 1st exon (70 CpG sites [12.37%]), 5′UTR (79 CpG sites [13.96%]), and were less from the regions like 3′UTR (10 CpG sites [1.77%]) and IGR (51 CpG sites [9.01%]). The right heatmap described the location of CpG sites: the detected DMPs were located in opensea (158 CpG sites [30.74%]), island (132 CpG sites [25.69%]), shore (123 CpG sites [23.93%]), and shelf (101 CpG sites [19.65%]). CpG sites of DMRs were in island (299 CpG sites [52.83%]), shore (123 CpG sites [23.93%]), and opensea (74 CpG sites [13.07%]) and were less located in shelf (4 CpG sites [0.71%]). **C** Bar plot for the top 10 terms for biological process, molecular function, and cellular component from gene ontology analysis of DMP-annotated genes. **D** Bar plot for the top 10 terms for biological process, molecular function, and cellular component from gene ontology analysis of DMR-annotated genes**. E** Venn diagram for the overlap gene between DMP and DMR. The illustrations of important DMPs were shown in (**F**–**K**), patients with GAD or OCD (case) and healthy controls (HC) are distinguished by different colors. The *Y*-axis represented the beta value of each CpG probe. Wilcoxon test was used to test the significance of difference of mean beta value between two groups. **L** The diagnose model performance in classification of cases and HC: the model got the AUC of 0.99 (95%CI 0.98–1, red line) and 0.90 (95%CI 0.86–0.96, blue line) in training dataset (*n* = 323) and test dataset (*n* = 137), respectively.

To investigate the epigenetic factors associated with the onset of GAD and OCD, we conducted the differential methylation analyses between patients with GAD and OCD (cases) and health controls (HC). We identified a total of 514 differentially methylated positions (DMPs), the top 10 significant DMPs were listed in Table 2, four of which were brain-blood correlated and were highlighted in bold. Compared to HC, 476 DMPs were hypermethylated and 38 DMPs were hypomethylated in cases (Fig. 1A). The regions of DMPs were shown in the left heatmap of Fig. 1B, about 43% of DMPs were enriched in promoter regions like TSS1500 (TSS, transcription start site; 100 CpG sites [19.46%]), TSS200 (43 CpG sites [8.37%]), 5’UTR (55 CpG sites [10.7%]), and 1st exon (23 CpG sites [4.47%]) and about 57% of DMPs were enriched in non-promoter regions like body (149 CpG sites [28.99%]), IGR (intergenic region, 123 CpG sites [23.93%]), and 3′UTR (21CpG sites [4.09%]). The locations of DMPs were shown in the right heatmap of Fig. 1B, that 30.74% (158 CpG sites), 25.69% (132 CpG sites), 23.93% (123 CpG sites), 19.65% (101 CpG sites) of DMPs were in opensea island, shore, and shelf, respectively. Gene ontology (Fig. 1C) revealed that DMP-affected genes involved the biological processes like the transduction and regulation of signaling, affected the cellular components like synapse which is pivotal for mental health disorders, and regulated the molecular functions like protein binding, enzyme binding, and DNA binding that are crucial for the regulation of transcription and expression of DNA. Besides, these genes were associated with the development process. Among the DMP genes, two CpG sites overlapped with the risk loci of GAD and OCD from published genome-wide association studies: cg10740573 (nearby *MTA1*, Fig. 1K) [18] and cg24646457 (nearby *PLA2G4D*, Fig. 1J) [19]. Among the top 10 DMP genes, we found two of them were related to mental health disorders: *RIOK3* (cg21515243, Fig. 1F) was a gender-specific risk factor for Alzheimer’s disease [20] and *PSMB4* (cg01334186, Fig. 1H) involved inflammation was related to the susceptibility to major depression [21]. Besides, *DNASE2* (cg09379601, Fig. 1G) was associated with the regulation of autoinflammation [22].

**Table 2:** Top 10 DMPs identified in the case-control study and between GAD and OCD.

Case-control study									GAD vs OCD study								
DMP	LogFC	*t*	*p*_*adj*_	CHR	BP	Gene	Region	Location	DMP	LogFC	*t*	*p*_*adj*_	CHR	BP	Gene	Region	Location
^a^cg21515243	7.2 × 10^−3^	8.22	8.0 × 10^−10^	18	21033072	*RIOK3*	1stExon	island	^a^cg21400344	−6.3 × 10^−2^	−9.61	4.4 × 10^−12^	1	25870172	*LDLRAP1*	5′UTR	Island
cg02895995	6.1 × 10^−3^	8.06	1.1 × 10^−9^	19	7554069	*PEX11G*	TSS200	shore	cg11027456	3.5 × 10^−2^	9.52	4.4 × 10^−12^	7	799693	*HEATR2*	Body	Shelf
^a^cg09379601	6.3 × 10^−3^	7.99	1.1 × 10^−9^	19	12992224	*DNASE2*	5′UTR	island	cg14509631	3.8 × 10^−2^	9.49	4.4 × 10^−12^	5	52694172	*/*	IGR	Opensea
cg03797768	6.8 × 10^−3^	7.98	1.1 × 10^−9^	13	24040813	*/*	IGR	island	cg20490126	−3.8 × 10^−2^	−9.14	2.8 × 10^−11^	1	25870007	*LDLRAP1*	TSS200	Island
^a^cg15094104	5.8 × 10^−3^	7.78	3.5 × 10^−9^	5	133406808	*/*	IGR	island	cg23712970	2.4 × 10^−2^	9.09	3.0 × 10^−11^	14	23540735	*ACIN1*	1stExon	Opensea
cg08052428	8.4 × 10^−3^	7.41	3.8 × 10^−8^	9	135996421	*RALGDS*	Body	island	^a^cg27272293	6.3 × 10^−2^	9.02	3.7 × 10^−11^	12	76531007	*/*	IGR	Opensea
cg01334186	6.9 × 10^−3^	7.04	3.7 × 10^−7^	1	151372572	*PSMB4*	Body	island	^a^cg25472897	−4.8 × 10^−2^	−8.93	5.6 × 10^−11^	8	145560555	*SCRT1*	TSS1500	Island
cg19613905	6.9 × 10^−3^	7	4.1 × 10^−7^	15	56757180	*MNS1*	1stExon	island	cg03862999	2.6 × 10^−2^	8.89	6.0 × 10^−11^	6	71874711	*/*	IGR	Opensea
cg08204369	7.1 × 10^−3^	6.82	1.2 × 10^−6^	17	73150733	*HN1*	5’UTR	island	^a^cg14502625	7.6 × 10^−2^	8.61	2.9 × 10^−10^	5	33162283	*/*	IGR	Opensea
^a^cg21607453	2.5 × 10^−3^	6.67	2.4 × 10^−6^	9	98783545	*NCRNA00092*	Body	island	^a^cg09608008	4.6 × 10^−2^	8.59	2.9 × 10^−10^	11	66529885	*C11orf80*	Body	Island

Abbreviations: *DMP* differentially methylated position, *logFC* log of fold-change, *t* t-statistic, *p*_adj_ adjusted *p* value, *CHR* chromosome, *BP* basepair, 5′*UTR* five prime untranslated region, *IGR* intergenic region, *TSS* transcriptional start site.

Annotation: ^a^, CpG site with significant brain-blood correlation.

We identified a total of 119 DMRs (constituted by 556 CpG sites) between cases and HC, most of them were brain-blood correlated. The top 10 significant DMRs were listed in Table 3 and showed blood-brain correlation. As shown in the left heatmap in Fig. 1B, in 556 CpG sites from DMRs, 73.15% of them were enriched in promoter regions including TSS1500 (130 CpG sites [22.97%]), TSS200 (135 CpG sites [23.85%]), 1st exon (70 CpG sites [12.37%]), and 5′UTR (79 CpG sites [13.96%]) and 26.85% of them were enriched in non-promoter regions including 3′UTR (10 CpG sites [1.77%]) and IGR (51 CpG sites [9.01%]). The right heatmap of Fig. 1B described the locations of these CpG sites: more than half of them were located (299 CpG sites [52.83%]) in island, 33.39% (189 CpG sites), 13.07% (74 CpG sites) and 0.71% (4 CpG sites) of them were in shore, opensea, and shelf, respectively. GO analysis indicated these DMR genes were related to the regulation of molecular functions like signaling and metabolism, cellular components like organelle, and biological processes like bindings of proteins and receptors (Fig. 1D). Same as we observed in DMPs, the DMR genes were involved in the development process such as nervous system development as well. Furthermore, we noticed that some DMR genes were associated with other mental health disorders as well. For example, the DMR gene *USP6NL* was a risk locus for Alzheimer’s disease [23, 24], and the DMR gene *CPLX1* was associated with cognitive resilience [25], nerve system development [26], and schizophrenia [27, 28]. The DMP and DMR overlapped 2 genes (Fig. 1E), one of them named *MEST* (Fig. 1I*)* was an imprinted gene whose DNAm level reflected the in-utero stress [29]. Full tables of DMPs, DMRs, and GO results can be found in Supplementary Tables 1–4.

**Table 3:** Top 10 DMRs identified in the case-control study and between GAD and OCD.

Case-control study						GAD vs OCD					
DMR	CHR	Start	End	*p*_adj_	Gene	DMR	CHR	Start	End	*p*_adj_	Gene
DMR_1	11	67383377	67384040	2.45 × 10^−4^	/	DMR_1	4	39183353	39183824	3.39 × 10^−3^	*WDR19*
DMR_2	3	107809536	107810507	2.74 × 10^−4^	*CD47*	DMR_2	6	28601312	28601519	2.35 × 10^−3^	/
DMR_3	6	28601312	28601519	5.28 × 10^−4^	/	DMR_3	11	842517	842615	2.19 × 10^−3^	*TSPAN4*
DMR_4	10	11504898	11505450	4.50 × 10^−4^	*USP6NL*	DMR_4	3	122513738	122514143	4.38 × 10^−3^	*DIRC2*
DMR_5	7	1022643	1022970	9.59 × 10^−4^	*CYP2W1*	DMR_5	10	33247232	33247545	3.08 × 10^−3^	*ITGB1*
DMR_6	2	27665079	27665150	6.95 × 10^−4^	*KRTCAP3*	DMR_6	16	75150611	75150880	3.93 × 10^−3^	*LDHD*
DMR_7	4	778827	779691	6.95 × 10^−4^	*CPLX1*	DMR_8	1	115397597	115398123	6.41 × 10^−3^	*SYCP1*
DMR_8	1	95698827	95699097	9.98 × 10^−4^	*RWDD3*	DMR_9	7	1022643	1022970	4.35 × 10^−3^	*CYP2W1*
DMR_9	1	24194974	24195659	1.01 × 10^−3^	*FUCA1*	DMR_10	5	93447204	93447700	5.74 × 10^−3^	*FAM172A*
DMR_10	1	152595322	152595992	1.16 × 10^−3^	*LCE3A*	DMR_12	1	32671304	32671884	6.70 × 10^−3^	*IQCC*

To evaluate whether our findings can diagnose the onset of GAD and OCD, we used the CpG sites identified in DMPs and DMRs and develop a diagnostic model. The diagnostic model showed robustness in the diagnosis of GAD and OCD with the AUC of 0.99(95%CI 0.98–1) and 0.90(95%CI 0.86–0.96) in the training dataset and test dataset, respectively (Fig. 1L).

### Differential methylation analyses between GAD and OCD

**Fig. 2:**
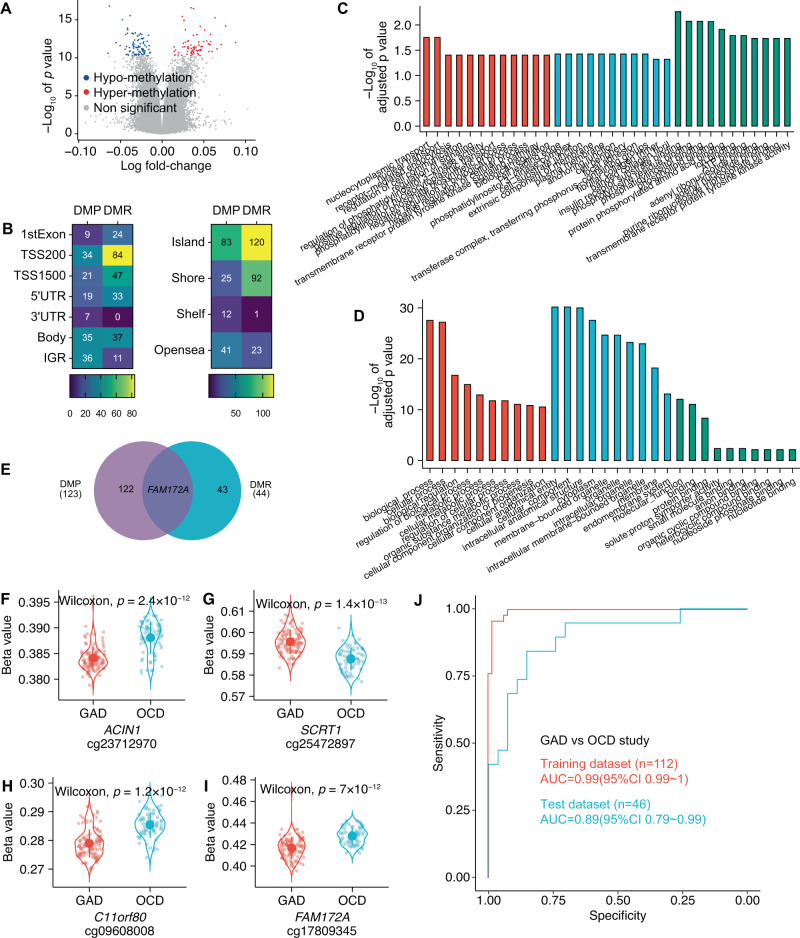
Differential methylation analyses between patients with GAD and OCD. **A** Volcano plot for differentially methylated positions (DMPs). Gray points represented the nonsignificant DMP; red and blue points represented the significant DMPs with hyper or hypo-methylation (GAD vs OCD), respectively. Between patients with GAD and patients with OCD, 161 significant DMPs were detected: **B** The heatmap plots. The left heatmap described that the detected DMPs were mainly enriched in regions as body (35 CpG sites [21.74%]), IGR (intergenic region, 36 CpG sites [22.36%]), and TSS1500 (21 CpG sites [13.04%]), TSS200 (34 CpG sites [21.12%]), and 5′UTR (19 CpG sites [11.8%]) and were less enriched in 3′UTR (7 CpG sites [4.35%]) and 1st exon (9 CpG sites [5.59%]) and that the CpG sites from DMRs were mainly from the regions as TSS200 (84 CpG sites [35.59%]), TSS1500 (47 CpG sites [19.92%]), 1st exon (24 CpG sites [10.17%]), 5′UTR (33 CpG sites [13.98%]), and body (33 CpG sites [13.98%]) and were less from the regions like IGR (11 CpG sites [4.66%]); the right heatmap illustrated that the detected DMPs were located in island (85 CpG sites [51.55%]), opensea (41 CpG sites [25.47%]), shore (25 CpG sites [15.53%]), and shelf (12 CpG sites [7.45%]) and that the CpG sites of DMRs were in island (120 CpG sites [50.85%]), shore (92 CpG sites [38.98%]), and opensea (23 CpG sites [9.75%]) and were less located in shelf (1 CpG site [0.42%]). **C** Bar plot illustrated the top 10 terms for biological process, molecular function, and cellular component from gene ontology analysis of DMP-annotated genes**. D** illustrated the top 10 terms for biological process, molecular function, and cellular component from gene ontology analysis of DMR-annotated genes. **E** Venn diagram for the overlap gene between DMP and DMR. The illustrations of important DMPs were shown in (**F**–**I)**, patients with generalized anxiety disorder (GAD) and patients with obsessive-compulsive disorder (OCD) are distinguished by different colors. The *Y*-axis represents the beta value of each CpG probe. Wilcoxon test was used to test the significance of difference of mean beta value between two groups. **J** The distinguishing model performance in classification of GAD and OCD: the model got the AUC of 0.99 (95%CI 0.99–1, red line) and 0.89 (95%CI 0.79–0.99, blue line) in training dataset (*n* = 112) and test dataset (*n* = 46), respectively.

We then investigated the DNAm alteration between GAD and OCD. As Fig. 2A illustrated, a total of 161 DMPs were detected between GAD and OCD. Compared to patients with OCD, 75 of them were hypermethylated, and 86 of them were hypomethylated in patients with GAD. Half part of the DMPs were enriched in promoter regions: TSS1500 (21 CpG sites [13.04%]), TSS200 (34 CpG sites [21.12%]), 5′UTR (19 CpG sites [11.8%]), and 1st exon (9 CpG sites [5.59%]); 21.74, 22.36, and 4.35% of DMPs were enriched in the gene body (35 CpG sites), IGR (36 CpG sites), and 3′UTR (7 CpG sites), respectively (Fig. 2B, left heatmap). Besides, 51.55% of DMPs were in island (85 CpG sites), 25.47, 15.53, and 7.45% of DMPs were in opensea (41 CpG sites), shore (25 CpG sites), and shelf (12 CpG sites), respectively (Fig. 2B, right heatmap). These DMPs were mapped into 123 DMP genes. In the top 10 DMP genes (Table 2, brain-blood correlated CpG sites were highlighted in bold), *ACIN1* (Fig. 2F) was associated with chronic stress [30], *SCRT1* (Fig. 2G) involved the conversion of microglia to neuron [31] and the nerve system development [32], *C11orf80* (Fig. 2H) was related to the embryo development [33]. GO analysis (Fig. 2C) revealed the DMP genes involved biological processes like transport, regulation of enzyme activity, and signaling, cellular components like cell membrane, junction, and adhesion, and molecular functions like bindings of receptors and substances.

We detected 53 DMRs (constituted by 236 CpG sites). The CpG sites of DMRs were largely (79.66%) from promoter regions including TSS200 (84 CpG sites [35.59%]), TSS1500 (47 CpG sites [19.92%]), 1st exon (24 CpG sites [10.17%]), and 5′UTR (33 CpG sites [13.98%]) and were less from the regions of gene body (33 CpG sites [13.98%]) and IGR (11 CpG sites [4.66%], Fig. 2B, left heatmap). Besides, 50.85% of them were in island (120 CpG sites) and the rest of them were in shore (92 CpG sites [38.98%]), opensea (23 CpG sites [9.75%]), and shelf (1 CpG site [0.42%], Fig. 2B, right heatmap). Same as case-control study, most of the CpG sites showed brain-blood correlation. Among the top 10 DMR genes (Table 3), *WDR19* was associated with ataxia [34], *SYCP1* regulated the meiosis progress [35, 36], *FAM173B* [37, 38] and *CCT5* [37] involved chronic pain. GO analysis indicated that DMR genes were associated with the metabolic and cellular process, organelles, and binding process (Fig. 2D). Overlap between DMP and DMR genes left gene *FAM172A* (Fig. 2H, L) that the DNAm of it was associated with the placental situation [39]. Full tables of DMPs, DMRs, and GO results can be found in Supplementary Tables 5–8, respectively.

In addition, we used the CpG sites identified in DMPs and DMRs to build a model and test its performance in distinguishing GAD from OCD (distinguish model). In the classification of GAD and OCD, the model performed robustly as the AUC of 0.99(95%CI 0.99–1) and 0.89(95%CI 0.79–0.99) in the training dataset and test dataset, respectively (Fig. 2J).

### Investigation of the epigenetic development trajectory

**Fig. 3:**
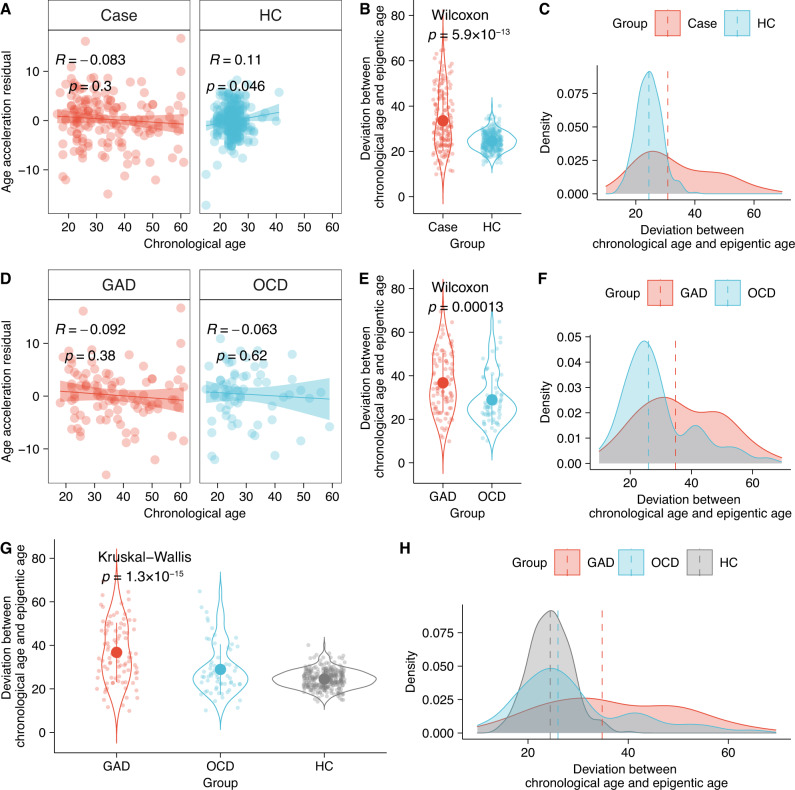
Estimation of the epigenetic clock (age acceleration difference). **A** Pearson correlation in the case-control study revealed a significant correlation (*p* < 0.05) between chronological age and epigenetic age (age acceleration residual) in health control (HC, right panel) and a nonsignificant correlation in cases (GAD and OCD, left panel). **B** Wilcoxon test indicated a significant difference of epigenetic-chronological deviation between cases and controls (*p* = 5.9 × 10^−13^). **C** Density plot for epigenetic-chronological deviation between cases and controls. **D** Pearson correlation of chronological age and epigenetic age (age acceleration residual) in GAD (left panel) and OCD (right panel), respectively. **E** Wilcoxon test indicated a significant difference of epigenetic-chronological deviation between GAD and OCD (*p* = 0.00013). **F** Density plot for epigenetic-chronological deviation between GAD and OCD. **G** Kruskal–Wallis test indicated a significant difference of epigenetic-chronological deviation between GAD, OCD, and HC (*p* = 1.3 × 10^−15^). **H** Density plot for the epigenetic-chronological deviation between GAD, OCD, and HC.

Results from DMP and DMR in both the case-control study and GAD vs OCD study indicated the DNAm alterations in genes that were associated with growth and development were in relation to the onset of and the divergence of GAD and OCD. Therefore, we estimated the age acceleration residual (epigenetic age), a measurement that reflects the epigenetic trajectory of growth and development, to compare the differences in development. Pearson correlation indicated a significant correlation between chronological age and epigenetic age in HC (*R* = 0.11, *p* = 0.046) but not in cases (Fig. 3A), which suggested a deviation of epigenetic age in patients compared to healthy controls. Besides, the direction of correlation in HC was positive whereas in cases was negative, which indicated the epigenetic age in cases might fall behind or overtake the chronological age. To further confirm the alteration in development trajectory, we compared the deviation of age, that is the difference between chronological age and epigenetic age, and found a significant difference in the deviation between cases and HC (Wilcoxon test, *p* = 5.9 × 10^−13^, Fig. 3B). The density plot showed that the median deviation in cases overtook the HC’s, which suggested epigenetic retardation in patients with GAD and OCD (Fig. 3C).

Then, we investigated the development trajectory between GAD and OCD. No significant correlation between chronological age and epigenetic age in both GAD and OCD (Fig. 3D). The Violin plot revealed a significant difference in the deviation between GAD and OCD (Wilcoxon test, *p* = 0.00013, Fig. 3E), which suggested a different development trajectory between subtypes of GAD and OCD. As the density plot shown in Fig. 3F, the median deviation of GAD overtook OCD’s, which indicated the speed of epigenetic development was slower in GAD than in OCD.

At last, we compared the deviation among GAD, OCD, and HC and found a significant difference among them (Kruskal-Wallis test, *p* = 1.3 × 10^−15^, Fig. 3G). The speed of epigenetic development was much closer to HC in OCD than in GAD (Fig. 3H)

## Discussion

In this study, we conducted epigenome-wide DNA methylation analyses in peripheral blood cells from 158 patients (93 patients with GAD and 65 patients with OCD) and 302 health controls of Chinese Han ancestry. Though the data we used were generated from blood tissue, our results showed that DMP and DMR genes were involved in the development and function of the central nervous system. Our results supported the important role of aberrant DNA methylation of neurodevelopmental genes in the onset and differentiation of GAD and OCD. Meanwhile, we found a different epigenetic trajectory that characterized GAD and OCD.

Mental health disorders such as schizophrenia, major depressive disorder (MDD), and bipolar disorder are polygenic inherited [40–42]. To date, two genome-wide association studies [18, 19] indicated the polygenic inheritance of GAD and OCD. However, whether GAD and OCD were polyepigenic inherited was not fully investigated. Our results identified aberrant DNA methylation across a total of 493 genes (383 DMP genes and 112 DMR genes), indicating the accumulative effects of DNA methylation on the pathogenesis of GAD and OCD. Besides, we identified aberrant DNA methylation in 166 genes (123 DMP genes and 44 DMR genes) that differentiated the GAD from OCD. These genes with aberrant DNA methylation, dysregulated the processes of metabolism, binding, and signaling and thus were involved in the onset and differentiation of GAD and OCD in different ways. Many of them showed brain-blood correlation in methylation level, suggesting the disease-related brain methylation status can be referred to the peripheral blood. To notice, the location where CpG sites were methylated has a different impact on gene expression. DNA methylation occurred in promoter regions (including 1st Exon, 5’UTR, TSS200, and TSS1500) can suppress the activity of transcription factors and then lead to expression reduction, whereas in gene body will upregulate the expression [43]. As our results described the CpG sites from DMPs and DMRs that were identified in both the case-control study and GAD vs OCD study were enriched in the promoter regions and gene body, thus the methylation changes may affect the gene expression. Considering the lack of matched transcriptome evidence, whether these methylation changes can regulate gene expression in real and involve the pathology should be explored in further study and be carefully demonstrated.

The presence of common genetic traits in mental health disorders has been demonstrated [44]. In the top of results from DMPs and DMRs, we found the shared mechanism among GAD, OCD, and other mental health disorders. For example, the DMP gene *RIOK3* and the DMR gene *USP6NL* (overlapped DMR gene between case-control study and GAD vs OCD study) were the risk genes for Alzheimer’s disease [20, 23, 24]. Anxiety-like feelings and compulsive behaviors can be seen in patients with MDD, schizophrenia, or cognitive disorders. The DMP genes *PSMB4* [21] had been reported associated with the susceptibility of MDD. The DMR gene *CPLX1* was aberrantly expressed in patients with schizophrenia [28] and was confirmed as the risk gene of schizophrenia [27]. Meanwhile, *CPLX1* involved cognitive resilience [25].

Studies indicate that GAD and OCD were development-related mental health disorders [4, 5]. We identified a set of genes that involved immune system development, cerebellar cortex development, and regulation of nervous system development. In the top of our results, the DMP gene *CPLX1* was associated with brain development and affected brain structure [26]. The DMP and DMR gene *MEST* that was identified in the case-control study was an imprinted gene associated with in-utero stress and can affect fetal development [29]. Between GAD and OCD, the differentially methylated genes we detected affected the development in different aspects: DMP gene *SCRT1* regulated the conversion from microglia to neuron which was crucial for nerve system development [31, 32]; DMR gene *SYCP1* regulated the fetal chromosome synapsis;[35, 36] DMP-DMR gene *FAM172A* was reported its DNA methylation was related to maternal circadian disruption which can affect the fetal development [39]. Through the epigenetic development trajectory analysis, we found the retardation of epigenetic development in patients with GAD and OCD compared to healthy controls and a different retardation level for each subtype, which supported the results of differential methylation analyses. Besides, neuroinflammation can be seen in patients with GAD or OCD. For example, the translator protein distribution volume (a neuroimaging biomarker reflecting microglia activation during neuroinflammation) was elevated in patients with OCD and prevention of the expression interleukin-33 in lipopolysaccharide-induced inflammation model attenuated the anxiety-like behavior. We identified two DMPs cg01334186 and cg09379601 nearby the inflammation-related genes *PSMB4* and *DNASE2*, respectively, were associated with the onset of GAD and OCD, which was supportive to the neuroinflammation hypothesis in GAD and OCD.

Both GAD and OCD can cause marked anxiety or distress in patients [2]. Investigation of the similarities and differences in epigenetics between the two diseases can contribute to the understanding of their mechanisms. Elevated serum cholesterol levels can be found in patients with GAD and OCD [45], we found *LDLRAP1* associated with hypercholesterolemia [46] was differentially methylated between GAD and OCD. Moreover, we discovered two genes *FAM173B* and *CCT5* from one DMR were associated with chronic pain [37]. However, there are fewer studies on pain in GAD and OCD, and there is a lack of sufficient evidence to clarify how this aberrant DNA methylation associated with chronic pain distinguishes GAD from OCD. A DMR gene *TNXB* associated with the onset of social anxiety disorder reported by Wiegand et al [47] was confirmed as a DMP gene for the onset of GAD and OCD in our study.

This study focused on DNAm change which is one of the epigenetic modifications. Besides it, other forms of epigenetic modifications like histone modification (e.g., phosphorylation, acetylation, ubiquitylation, and sumoylation) and non-coding RNA (e.g., micro RNA) involved OCD and GAD, respectively. Abnormal phosphorylation was associated with repetitive behaviors (RBs) which are the signature of OCD, for example, the elevated phosphorylation of rpS6 (neural activity marker) promoted RB in mice [48] and abnormal phosphorylation of RAP1 can be found in patients with OCD [49]. It can affect anxiety-like behavior as well that the study reported the phosphorylation levels of ERK (extracellular signal-regulated kinase) in the amygdala were associated with anxiety symptoms in human beings [50]. Studies suggested the influences of acetylation and sumoylation in anxiety-like behaviors, for example, increased acetylation of Arc SARE attenuated anxiety-like behavior in adult rats, besides, sumoylation of Rac1 [51] and serotonin 1a receptor [52] regulated anxiety-like behavior. Micro RNAs (miR) were reported to regulate the related symptoms of GAD or OCD as well. The physiological pathways of anxiety associated with the expression of BDNF, HTR2C, MAOA, and RGS2 can be affected by miR-22 [53] and the repetitive behavior in heterozygous knocked-out mice can be prompted by the partial loss of miR-137 [54]. These forms of epigenetic modifications of the genes identified in our study were not reported, which suggested the DNAm may be one unique epigenetic modification for the pathology of GAD or OCD.

Since the first time Horvath developed the epigenetic clock and observed different developmental trajectories between patients with cancers and healthy people [16], people realized the gap between epigenetic age and chronological age may reflect the pathology of diseases. The epigenetic developmental trajectories in patients with psychiatric disorders were abnormal compared to healthy controls and varied among different psychiatric disorders. For example, the epigenetic clock was retarded in patients with schizophrenia [55] and was accelerated in patients with posttraumatic disorder [56], bipolar disorder [57], or major depressive disorder [58]. The epigenetic clock among GAD and/or OCD was less investigated, and our results add the understanding of the epigenetic characteristics of GAD and OCD in a further step.

Disease-related DNA methylation changes could serve as biomarkers to assess, diagnose, and monitor disease [59, 60]. Our findings yielded two models that were robust in diagnosing and differentiating GAD and OCD. Though sample size of our study provided sufficient statistical power, validation in a larger sample size and different ethics for the models would be required in future study for their generalization. Ultimately, our results showed the promise of DNA methylation in identifying patients from healthy controls or classifying patients with GAD and OCD, which can be a precision medicine implementation and may aid in early detection, accurate diagnosis, and individualized treatment of GAD and OCD.

## Supplementary information

Supplementary Figure S1 | Statistical power of the sample size.

Supplementary table 1 | Differentially methylated positions (DMPs) identified in the case-control study

Supplementary table 2 | Gene ontology of DMP genes identified in the case-control study

Supplementary table 3 | Differentially methylated regions (DMRs) identified in the case-control study

Supplementary table 4 | Gene ontology of DMR genes in the case-control study

Supplementary table 5 | Differentially methylated positions (DMPs) identified between GAD and OCD

Supplementary table 6 | Gene ontology of DMP genes identified between GAD and OCD

Supplementary table 7 | Differentially methylated regions (DMRs) identified between GAD and OCD

Supplementary table 8 | Gene ontology of DMR genes identified between GAD and OCD
