# Supplementary material for: Epigenome-wide DNA methylation analysis of whole blood cells derived from patients with GAD and OCD in the Chinese Han population
Source: Transl Psychiatry. 2022 Nov 7;12:465. doi: 10.1038/s41398-022-02236-x (PMC9640561; doi:10.1038/s41398-022-02236-x)
Supplement: Supplementary file 1 — Supplementary Figure S1 | Statistical power of the sample size. [file 41398_2022_2236_MOESM1_ESM.docx]

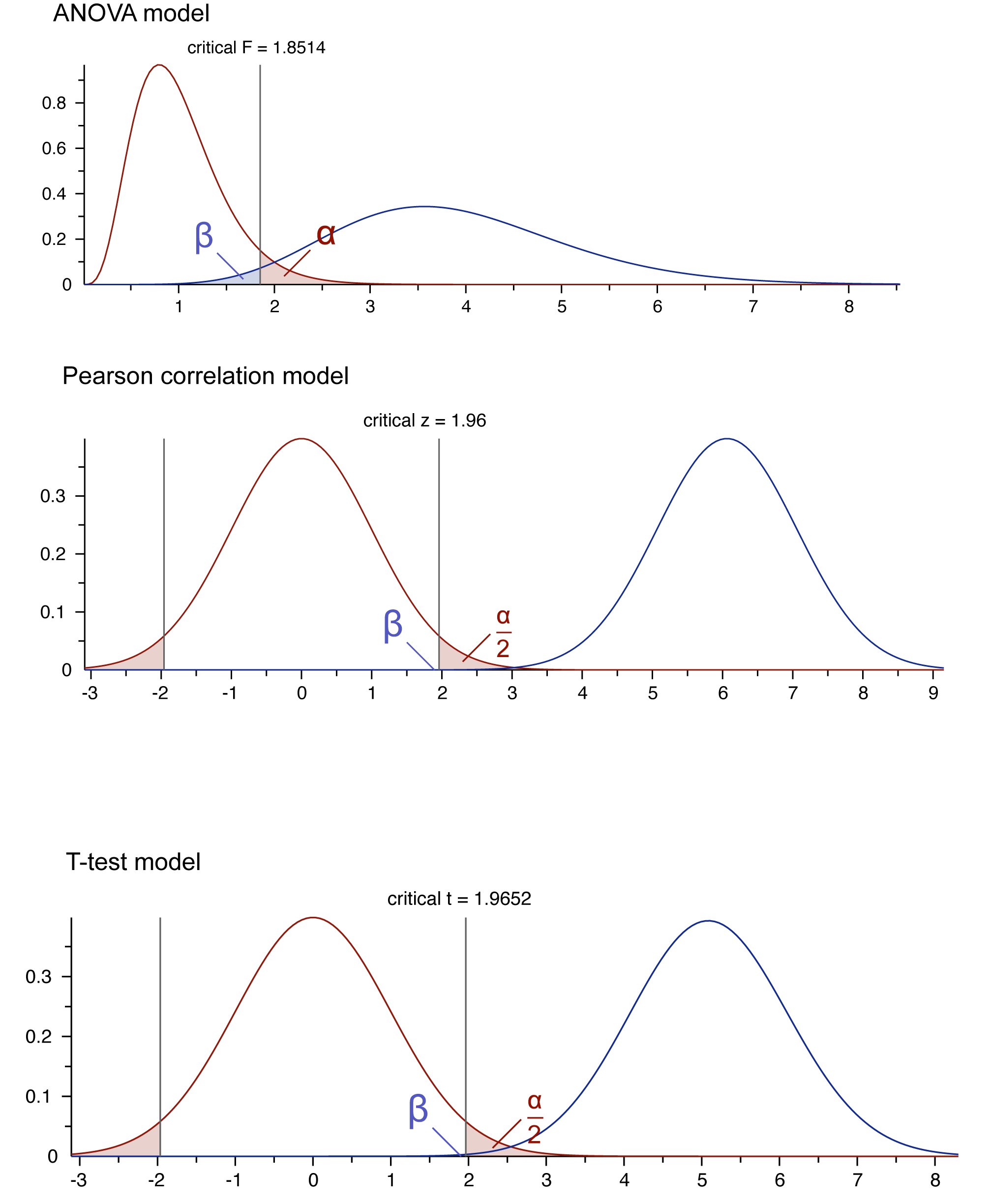


**Supplementary Figure S1**

The sample size of our study showed sufficient statistical power (1-β_error probability_ > 0.9) under the models of Pearson correlation, ANOVA, and t-test.
